# An Unusual Case of Chronic Partial Quadriceps Tear in a Child: A Case Report

**DOI:** 10.31729/jnma.5224

**Published:** 2020-12-31

**Authors:** Raymond D.K. Yeak, Y.Y. Yap, Nasir M Nizlan

**Affiliations:** 1 Department of Orthopaedic Surgery, Faculty of Medicine and Health Sciences, Universiti Putra Malaysia, 43400 Serdang, Selangor, Malaysia; 2 Department of Haematology, Ampang Hospital, Jalan Mewah Utara, Pandan Mewah, 68000 Ampang, Selangor, Malaysia

**Keywords:** *child*, *rupture*, *prognosis*, *tendon injuries*

## Abstract

Quadriceps tendon rupture usually occurs in adults and is rare in children. A six-year-old boy was playing at home and had a fall. He was unable to extend his right knee but there was no gap felt over the patella tendon or quadriceps. He was first seen by a family doctor and presented late to the surgeon three months after the injury. Radiographs and ultrasound were performed. The magnetic resonance imaging confirmed the findings of partial quadriceps tear. The patient was put in a cylinder case with the knee in extension for two months. Six months post-injury, he regained full range of motion without any complications. We present an unusual case of partial quadriceps tear in an otherwise healthy six-year-old boy that was treated successfully despite a delayed presentation. Besides a high index of suspicion, magnetic resonance imaging is a good modality to detect partial quadriceps tear in children.

## INTRODUCTION

This case report highlights a critical and rare observation that requires a high index of suspicion and was treated successfully. In this manuscript, we show that the unusual case of partial quadriceps tear in a healthy six-year-old boy can be treated successfully despite its delayed presentation. It highlights the disease mechanism as well as the need for critical clinical observation.

## CASE REPORT

**Figure 1:**
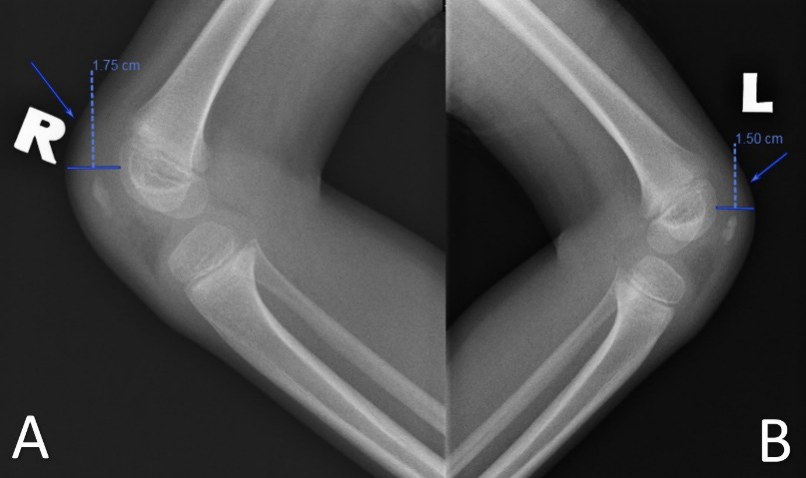
Right (A) and left (B) knee lateral radiographs and the marked area (arrow) of suprapatellar soft tissue swelling and the measurements.

A six-year-old boy with no known medical illness was playing at home when he attempted to jump onto the stool but ended up falling with the knee flexed and landed on the anterior aspect of his right knee. He then complained of pain associated with swelling over the right knee. He was able to flex his knee but unable to extend his right knee. On physical examination, there was no gap felt over the patella tendon or quadriceps but there was tenderness over the superior pole of the patella. He was first seen by a family doctor who treated him as having a soft tissue injury. The patient was only diagnosed after being seen by an orthopedic surgeon three months post-trauma. The radiograph of the right knee showed soft tissue swelling over the suprapatellar bursa (Figure 1).

**Figure 2:**
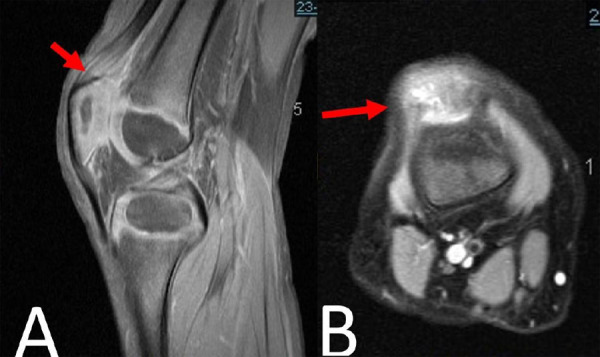
MRI in the coronal (A) and axial (B) section showing the high signal intensity (arrow) of the right knee highlighting the partial quadriceps tear just proximal to its patellar insertion.

A bedside ultrasound was done which showed a hypoechoic gap and quadriceps tear was noted. Magnetic resonance imaging (MRI) was then performed and the patient was diagnosed to have partial quadriceps tear (Figure 2).

The patient was then put in a cylinder cast with the knee in extension for two months. Full weight-bearing was allowed. Range of motion exercises was started immediately after removal of the cast. At his follow-up one month (six months post-trauma) later, the patient regained full range of motion without any complications.

## DISCUSSION

Quadriceps tendon rupture usually occurs in middle-aged to elderly men, especially those with an underlying chronic illness such as renal failure, gout, and diabetes mellitus.^1^

It is difficult for healthy tendons to rupture without an underlying inflammatory disease, degenerative change, or repetitive steroid injections.^2^

Rupture of the quadriceps tendon in the pediatric age group is extremely rare.^3^ This is because children are more prone to sustain an avulsion injury rather than a tendon injury due to the strength of the muscle-tendon unit in a child where the weakest link is the apophysis.^4^ We report three months delayed presentation of a six-year-old healthy boy who presented with a partial rupture of the quadriceps muscle after a fall. The tear also occurred just proximal to its patella insertion which corresponds to other cases that occur 1-2 cm from the avascular region of the quadriceps of the upper patellar pole.^5^

Kelly et al reported that quadriceps tendon ruptures are usually partial, involving mainly the rectus femoris tendon or its insertion and the prognosis should be more guarded.^2^

There have been case reports of partial tears that were successfully treated conservatively.^3,6^ However, to the best of our knowledge, there have never been any reports of chronic partial quadriceps tear in a child that was successfully treated conservatively.

Besides the history and physical examination, ultrasound and MRI were also helpful in determining the diagnosis. Ultrasound is a popular imaging modality due to its lower cost, greater availability, and dynamic imaging with no contraindications unlike MRI.^7^ However, MRI has a higher sensitivity in follow-up imaging especially in detecting ongoing muscle healing and in differentiating between old and new lesions.^7,^8^^ MRI is also not user-dependent unlike ultrasound.^7^ Therefore, MRI helps determine the extent of injury especially in partial tears.

The MRI showed a high signal intensity of the right knee highlighting the partial quadriceps tear just proximal to its patellar insertion. The surrounding edema and hemorrhage at three months could be due to reinjury at the site as the child was not treated or immobilized.^9^ We can then plan our surgical or conservative treatment based on the findings. In this case, the patient was treated conservatively.

We present an unusual case of chronic partial quadriceps tear in an otherwise healthy six-year-old boy that was treated successfully despite a delayed presentation. A high index of suspicion is needed to diagnose the condition and MRI is a good modality to detect partial quadriceps tear in children.
